# Oxygen activation in NO synthases: evidence for a direct role of the substrate

**DOI:** 10.1002/2211-5463.12036

**Published:** 2016-03-18

**Authors:** Albane Brunel, Jérôme Lang, Manon Couture, Jean‐Luc Boucher, Pierre Dorlet, Jérôme Santolini

**Affiliations:** ^1^ Laboratoire Stress Oxydant et DétoxicationInstitute for Integrative Biology of the Cell (I2BC)CEA, CNRS, Université Paris‐SaclayGif‐sur‐Yvette CedexFrance; ^2^ Département de biochimie, de microbiologie et de bio‐informatique, and PROTEOPavillon Charles‐Eugène MarchandUniversité LavalQuébecCanada; ^3^ UMR 8601 CNRS‐University Paris DescartesParisFrance

**Keywords:** kinetics, mechanism, nitric oxide synthase, oxygen activation, resonance Raman spectroscopy

## Abstract

Nitric oxide (NO) and the other reactive nitrogen species (RNOS) play crucial patho‐physiological roles at the interface of oxidative stress and signalling processes. In mammals, the NO synthases (NOSs) are the source of these reactive nitrogen species, and so to understand the precise biological role of RNOS and NO requires elucidation of the molecular functioning of NOS. Oxygen activation, which is at the core of NOS catalysis, involves a sophisticated sequence of electron and proton transfers. While electron transfer in NOS has received much attention, the proton transfer processes has been scarcely investigated. Here, we report an original approach that combines fast‐kinetic techniques coupled to resonance Raman spectroscopy with the use of synthetic analogues of NOS substrate. We characterise Fe^II^‐O_2_ reaction intermediates in the presence of L‐arginine (Arg), alkyl‐ and aryl‐guanidines. The presence of new reaction intermediates, such as ferric haem‐peroxide, that was formerly postulated, was tracked by analysing the oxygen activation reaction at different times and with different excitation wavelengths. Our results suggest that Arg is not a proton donor, but indirectly intervenes in oxygen activation mechanism by modulating the distal H‐bond network and, in particular, by tuning the position and the role of the distal water molecule. This report supports a catalytic model with two proton transfers in step 1 (Arg hydroxylation) but only one proton transfer in step 2 (N^ω^‐hydroxy‐L‐arginine oxidation).

**Abbreviations**
4‐CF_3_‐phgua: 4‐trifluoromethyl‐phenyl‐guanidine
4‐Clphgua: 4‐chlorophenyl‐guanidine
4‐Fphgua: 4‐fluorophenyl‐guanidine
4‐MeOphgua: 4‐methoxyphenyl‐guanidine
Arg: L‐arginine
BH_2_: dihydrobiopterin
BH_4_: tetrahydrobiopterin, (6R)‐5,6,7,8‐tetrahydro‐L‐biopterin
Cpd I: Compound I in chloroperoxidase, cytochrome P450 and catalase catalytic cyle
iNOS: inducible nitric oxide synthase
NO: nitric oxide
NOHA: N^ω^‐hydroxy‐L‐arginine
NOS: nitric oxide synthase
NOSoxy: oxygenase domain of NOS
NOSred: reductase domain of NOS
Pentylgua: *n*‐pentyl‐guanidine
RR: resonance Raman

Nitric oxide (NO) synthases were first characterised in mammals in the early 1990s as the source of NO in mammalian cells. Constitutive NO synthases (NOSs) are involved in signalling processes such as blood pressure regulation and angiogenesis (eNOS) 1 or learning process, synaptic plasticity and neurotransmission (nNOS) 2. The inducible NOS (iNOS) is recruited for nonspecific immune response against tumours, viruses or bacteria 3. On the other hand, NOSs have been increasingly associated with the development of several pathologies including cardiovascular, inflammatory and neurodegenerative diseases, diabetes and cancers 4, 5, 6, 7. Despite a broad range of biological activities, mammalian NOSs share a similar 3D structure and a common catalytic mechanism. NOSs synthesise NO in two stages 8: the first haem‐based oxidation converts L‐arginine (Arg) to a stable intermediate, N^ω^‐hydroxy‐L‐arginine (NOHA), and the second converts NOHA to L‐citrulline and NO 9, 10. Mammalian NOSs are homodimers that contain an N‐terminal oxygenase domain (NOSoxy) and a C‐terminal reductase domain (NOSred). Electron transfer between the two domains is regulated by a Calmoduline‐binding interface. NOSoxy binds Arg, the haem cofactor and the redox‐active cofactor tetrahydrobiopterin (BH_4_). NOSred is similar to P450 reductase and thus contains an NADP(H)‐ and FAD‐binding flavodoxin‐reductase module and a flavodoxin‐like FMN‐binding domain 8, 11, 12. Due to the high biomedical importance of mNOSs, their molecular mechanism has attracted tremendous interest. The first O_2_ activation step (Step 1) is similar but distinct from that of the cytochromes P450 (Fig. 1) 13: NOS catalysis begins with the reduction in the high‐spin ferric haem (Fe^III^) to ferrous haem (Fe^II^) by NOSred (with electrons originating from NADPH). Subsequent O_2_ binding forms the ferric superoxo‐haem (Fe^III^‐OO°) 14, which is further reduced by BH_4_ and protonated to form the ferric hydroperoxo‐haem (Fe^III^‐OOH) 15, 16, 17, 18. The hydroperoxo‐haem is thought to collapse to a Compound I intermediate (Cpd I: Fe^IV^(O)‐π^•+^), which then hydroxylates Arg. The BH_4_^•+^ radical is then re‐reduced by NOSred 19. In the second stage (Step 2), product formation requires only one electron, whereas oxygen activation still requires two electrons. The hydroperoxo intermediate, once formed, directly reacts with NOHA to eventually produce L‐citrulline and NO. In this stage, the second electron needed for oxygen activation may be shuttled back to the BH_4_^•+^ radical from a transient redox intermediate, which may be nitroxyl (NO^−^) 20.

**Figure 1:**
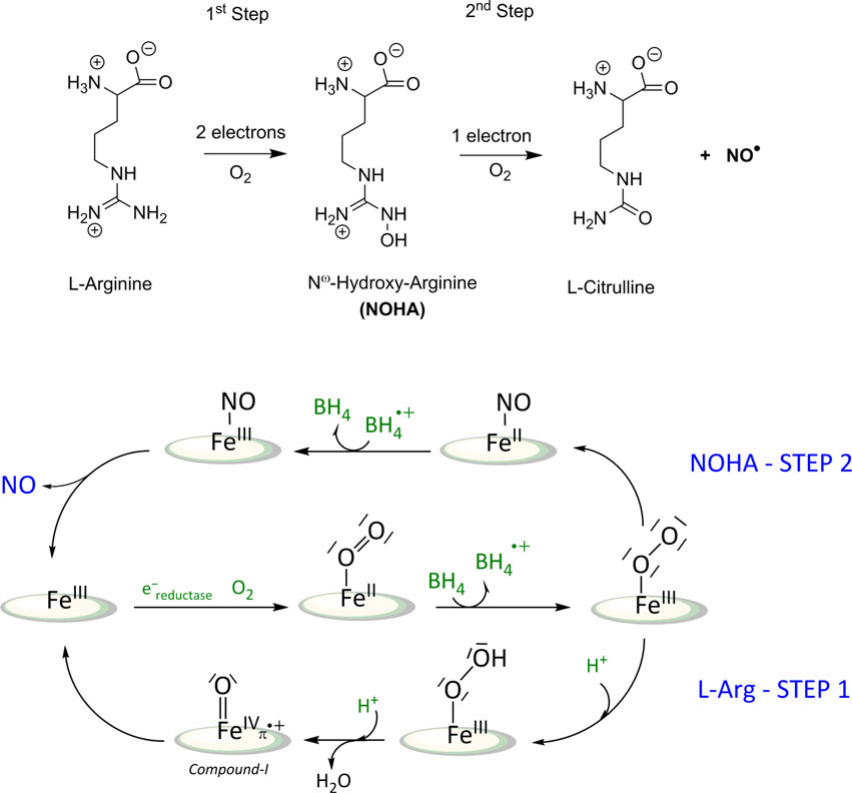
Catalytic and molecular mechanism of NOS. A: The two oxidative steps that sequentially convert the guanidinium moiety of Arg into NOHA and L‐citrulline with NO as side‐product. B: Current model of NO‐Synthase molecular mechanism. So far, only the initial and final intermediates (Fe^II^‐O_2_ and Fe^III^‐NO/Fe^III^) have been trapped and characterised in catalytic conditions. The key catalytic intermediates (haem‐peroxo, oxoferryl and ferrous haem‐NO complexes) remain yet to be identified.

However, this sequence remains essentially hypothetical. The only reaction intermediate that has been identified so far, besides the oxygenated complex, is the haem‐peroxide species, and only through cryo‐reduction approaches 21, 22 that do not mimic catalytic conditions. There is also no clear understanding of the reason of the coexistence of two distinct mechanisms for the two catalytic steps: if the first step of O_2_ activation is similar to what has been proposed for cytochromes P450, the second step is specific to NOS. Many research programmes have investigated two major specificities of NOS catalysis, which is the electron transfer by the BH_4_ cofactor 23, 24, 25 and the push effect of the proximal ligand 26, 27, 28. However, only scarce attention has been devoted to the proton transfer process (nature of the proton donor, sequence and pathway of proton transfer). Proton transfer is also at the heart of NOS molecular mechanism for it controls the balance between Arg hydroxylation (two protons) and NOHA oxidation (one proton), and tunes the coupling between electron transfer and oxygen activation, preventing NOS uncoupling and ROS production.

There are only few data on proton transfer and no comparative investigation of Steps 1 and 2. This is mostly due to the instability of the Fe^II^‐O_2_ intermediate, which requires often the use of noncatalytic conditions (redox analogues, low temperature…). However, some reports have addressed the question of proton transfer and/or the protonation of haem‐oxo species 13: among them, a large series of works have established the influence of the distal environment on the electronic structure, stability and reactivity of Fe^II^‐O_2_, ‐CO and ‐NO species 29, 30, 31, 32, 33. X‐ray and Raman data 29, 32, 34, 35, 36 have clearly shown that the H‐bond distal network undergoes a rearrangement as a function of the nature of the substrate (Arg, NOHA, analogues), with changes in the interaction between the substrate guanidinium and the distal ligand and in the role of a vicinal water molecule. We utilised a series of Arg analogues that were distributed along three groups according to their influence on the distal H‐bond network as determined from the spectroscopic signature of the CO complex probed with resonance Raman (RR) spectroscopy 33: Family 1 analogues had an effect similar to that of L‐Arg, Family 2 similar to NOHA and Family 3 corresponded to analogues that bind the active site but do not induce a spectroscopic signature, thus mimicking the absence of substrate. We showed that these changes modify the reactivity of the Fe^II^‐O_2_ species 33, 37 and the fate of the ferric haem‐peroxo intermediate: in the presence of Arg, the oxo ligand would be H‐bonded to the water molecule, favouring the O‐O heterolytic cleavage (and the build‐up of a Cpd I‐like species), whereas the direct H‐bond between NOHA guanidinium and the peroxo species would favour its stability and its direct reactivity 33. This indicates that the difference between Steps 1 and 2 depends on the structure of the distal H‐bond network: the distal water molecule would therefore be required for the first catalytic step (Arg hydroxylation) but not for the second one (NOHA oxidation) 13, 33. This has been confirmed by the work of Marletta and coworkers with 5‐methyl Arg and NOHA analogues 38.

This model has been mostly thought of from data using Fe^II^‐O_2_ mimics such as the stable Fe^II^‐CO and Fe^II^‐NO complexes. However, these species present structural and electronic properties different from those of Fe^II^‐O_2_. To overcome the instability of Fe^II^‐O_2_, that prevents its direct investigation, we have used here two complementary fast‐kinetics approaches: (a) the stopped‐flow set‐up coupled to UV–vis spectrometry allows determining the rate constants of O_2_ activation and autoxidation; (b) the T‐mixer set‐up coupled to RR spectroscopy allows characterising the structural properties of Fe^II^‐O_2_ intermediate and identifying short‐lived intermediates undetected by UV–visible spectroscopy.

We used this approach on one hand to investigate the effect of Arg analogues on the Fe^II^‐O_2_ complex and verify the actual role of the water molecule in NOS catalysis, and on the other hand to trap and characterise new reaction intermediates that may intervene in Arg hydroxylation.

## Experimental procedures

### Chemicals

**Figure 2:**
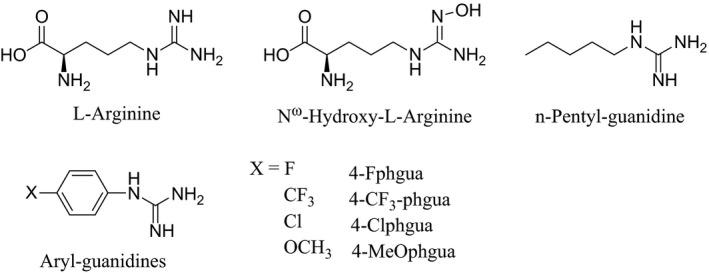
Structure of *n*‐pentylguanidine and aryl‐guanidines studied in this work.

The synthesis of the hydrochloride salts of *n*‐pentyl‐guanidine (Pentylgua), 4‐fluorophenyl‐guanidine (4‐Fphgua), 4‐chlorophenyl‐guanidine (4‐Clphgua), 4‐trifluoromethyl‐phenyl‐guanidine (4‐CF_3_‐phgua) and 4‐methoxyphenyl‐guanidine (4‐MeOphgua) (Fig. 2) have been described elsewhere 37. BH_4_ and Arg were purchased from Enzo Life Sciences (Enzo Life Sciences, Farmingdale, NY, USA)

### Enzyme preparation

Mouse iNOS oxygenase domain (iNOSoxy) containing a six‐histidine tag at its C‐terminus was expressed in *Escherichia coli* BL21 using the PCWori vector and purified as already described 39 in the absence of BH_4_ and Arg.

### Stopped‐flow experiments

The protein samples containing BH_4_/BH_2_ were obtained by preincubation at 4 °C in the presence of 200 μm BH_4_/400 μm BH_2_ and 5 mm Arg or 10 mm of compounds Pentylgua and 4‐Fphgua in 100 mm Kpi buffer (pH 7.4). The binding of the guanidine derivatives and BH_4_ was confirmed by measuring the low‐spin (420 nm) to the high‐spin (395 nm) transition of the haem using UV–vis spectroscopy. The samples were made anaerobic by several cycles of vacuum and argon refilling. The reduction in the haem was achieved by progressive addition of an anaerobic solution of sodium dithionite and followed by monitoring the change in the Soret peak from 395 nm for the ferric form to 412 nm for the ferrous one. The rapid‐mixing stopped‐flow experiments were performed at 4 °C on a Bio‐Logic SFM 300 instrument customised for anaerobic and semianaerobic experiments and connected to a Tidas 1024‐diode array detector able of recording spectra every 3 ms. The reduced enzyme was transferred into the stopped‐flow apparatus with a gastight syringe and was rapidly mixed with an equal volume of air saturated buffer. Spectra were recorded between 350 and 700 nm with a total measurement time between 2 and 5 s.

### Stopped‐flow data analysis

We used a direct examination of the recorded spectra with the absorbance cross sections at specific wavelengths performed with originpro 8.0 software (OriginLab Corporation, Northampton, MA, USA). First, visual analysis of the spectra with BH_2_ allowed us to identify the different haem species involved in the single‐turnover reaction: ferrous Fe^II^ (412 nm), ferrous dioxygen Fe^II^‐O_2_ (427–430 nm) and ferric Fe^III^ (395 nm) complexes.

The determination of quantitative transition rates were obtained by combining different methods. The autoxidation rates (in the presence of BH_2_) reported by Moreau *et al*. 37 were determined by global analysis (SPECFIT) and by simulation of time traces at 428/395/650 nm to a mono‐exponential function. Rate constants obtained by these different methods were similar for every condition 37. In this report, autoxidation rates were obtained by global analysis like Moreau *et al*. 37 but also by simulation of the 428 nm time trace to a biexponential function. Here again, rates obtained via these two methods were similar.

Activation rates (in the presence of BH_4_) were all obtained like Moreau *et al*. 37, that is, by simulation of the 395 nm time traces to a single mono‐exponential function, except in the presence of Arg and 4‐methoxyphenyl‐guanidine, for which we simulated the kinetics to a biexponential function.

### Resonance Raman spectroscopy of iNOSoxy oxygenated intermediates

The buffer used for kinetic measurements was 100 mm KPi, pH 7.4. To prepare the ferrous form of iNOSoxy, the ferric enzyme (80 μm) was equilibrated with nitrogen gas for 30 min at room temperature, and the haem was then reduced with sodium dithionite. Complete reduction in iNOSoxy was verified by optical spectroscopy. Oxygen‐containing buffer solutions were prepared by equilibrating deoxygenated buffer with ^16^O_2_ or ^18^O_2_. The rapid T‐mixer used here was described previously 40. Oxygen was removed from the mixer with an anaerobic buffer prior to connecting the syringes containing the ferrous protein and oxygen‐saturated buffer. The output at 413.1 nm is from a Kr ion laser (Innova 302 Kr; Coherent, Santa Clara, CA, USA) at 10 mW. The output at 441.6 nm is from a He/Cd ion laser (Liconix Laser; Melles‐Griot, Ottawa, ON, Canada) at 10 mW. The position of the laser‐focusing point was moved along the flow direction to obtain the desired time point. The flow rate was adjusted to result in a time point of 1.25 ms mm^−1^. The data were measured at time delays after mixing ranging from 4 to 40 ms and measurements were recorded at room temperature (25 °C). The RR spectra were calibrated with the signals from indene. The RR spectrum of reduced myoglobin was recorded prior to each mixing experiment and was used to adjust small differences in the calibration of spectra from different mixing experiments.

## Results

### Kinetic analysis of Fe^II^‐O_2_ activation and autoxidation reactions

We first investigated the effect of Arg and various aryl‐guanidines on the kinetics of oxygen activation and of Fe^II^‐O_2_ autoxidation. This work aimed at completing the results we previously obtained 33, 37 for supplemental compounds of Family 2 and 3 analogues (Fig. 2) in order to determine the best conditions for the T‐mixing experiments observed by RR spectroscopy, in particular the optimal build‐up times. We used the same stopped‐flow methods and the same kinetic analysis 33, 37 (see Experimental procedures) to determine the rates of Fe^II^‐O_2_ autoxidation in the presence of BH_2_ (a redox‐inactive BH_4_ analogue) and the rates of oxygen activation, in the presence of BH_4_.

**Figure 3:**
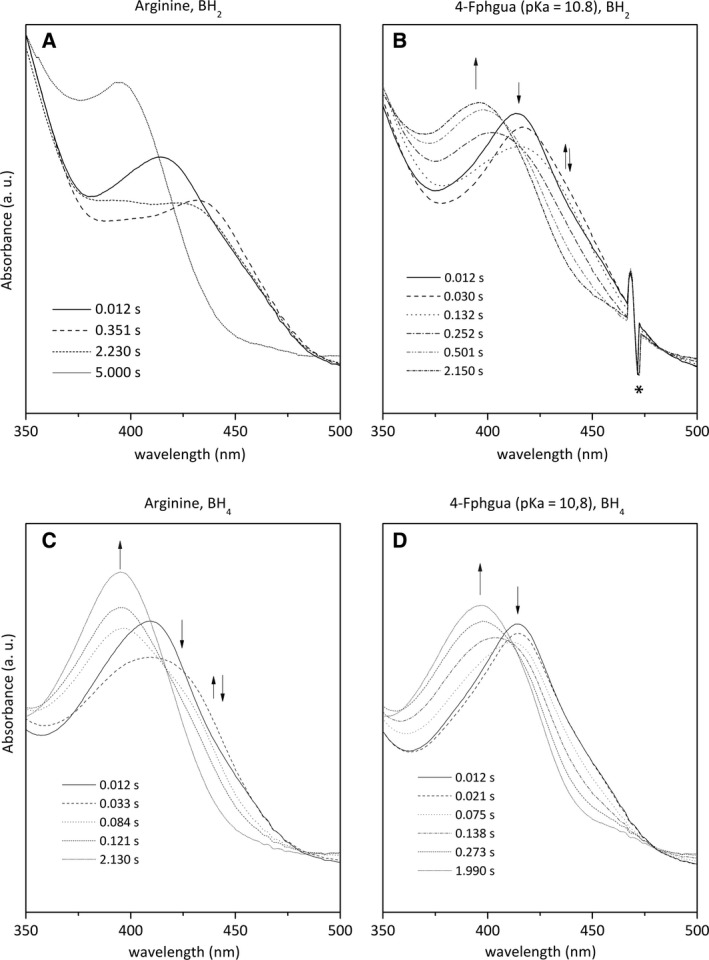
Absorbance spectra for the reaction of ferrous iNOSoxy with oxygen, in the presence of BH_2_ (A, B) or BH_4_ (C, D) and L‐Arg (A, C) or 4‐Fphgua (B, D). The enzyme was rapidly mixed at 4 °C with an equal volume of oxygen‐saturated buffer (see Experimental procedures). Solid lines correspond to the spectra of the initial reduced forms; dashed and dotted lines correspond to the spectra at various times after rapid‐mixing.

**Figure 4:**
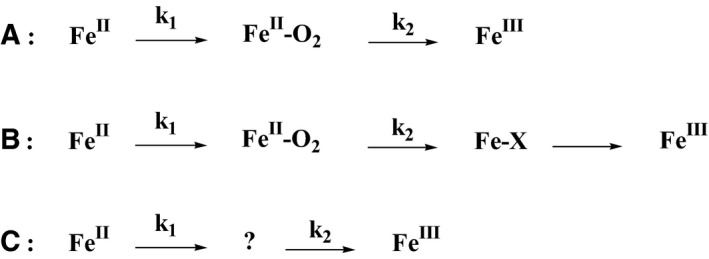
Reaction sequences analysed by optical stopped‐flow spectroscopy and RR spectroscopy with the T‐mixer. A: Autoxidation reaction (in the presence of BH_2_). Rates between 0.2 and 19 s^−1^ 33, 37. B: Oxygen activation reaction (in the presence of BH_4_). Rates of the ferric heme recovery around 20 s^−1^ 33, 37. C: Oxygen activation reaction in the presence of Family 2 analogues. Rates of the ferric heme recovery are smaller than those of Fe^II^‐O_2_ build‐up 33, 37.

In the presence of BH_2_, we observed the same Fe^II^ → Fe^II^‐O_2_ → Fe^III^ transition in the presence of Arg (Fig. 3A) or various aryl‐guanidines analogues (Fig. 3B). The rates of Fe^II^‐O_2_ decay were determined by simulating the time traces at 427 nm (Fe^II^‐O_2_ formation and decay) and at 395 nm (Fe^III^ recovery) with a biexponential function (see Experimental procedures). Our results confirm that changes in the substrate does not modify oxygen‐binding rate (k_1_) but that autoxidation rates (k_2_) successively increase for Family 1 and Family 2/3 analogues, as already described 37 (Fig. 4A, Table 1). In the presence of BH_4_, the same sequence of intermediates is observed (Fig. 4B) with a smaller build‐up of the Fe^II^‐O_2_ species (Fig. 3C,D), illustrating the oxygen activation upon an electron transfer from BH_4_, as already reported 41, 42, 43. The kinetics analysis confirms the absence of Fe^II^‐O_2_ build‐up in the presence of aryl‐guanidines analogues, as previously reported 41, 42, 43. The moderate increase in the rate of Fe^III^ recovery (between 12% and 100%, Table 1) should not have prevented observing the build‐up of this intermediate (Fig. 4C), suggesting the existence of a transient reaction intermediate that cannot be observed by conventional UV–visible spectrometry, as already proposed 37.

**Table 1:** Determination of autoxidation (BH_2_) and activation rates (BH_4_) in the presence of Arg and various Arg analogues

		Arg (pKa = 12.5)		Family 1 Pentylgua (pKa = 12.6)		Family 2 4‐Fphgua (pKa = 10.8)		Family 2 4‐CF_3_‐phgua (pKa = 10)		Family 2 4‐Clphgua (pKa = 10.3)		Family 3 4‐MeOphgua (pKa = 11)	
		BH_2_	BH_4_	BH_2_	BH_4_	BH_2_	BH_4_	BH_2_	BH_4_	BH_2_	BH_4_	BH_2_	BH_4_
This report	k_1,_ s^−1^	> 50	> 50	nd	nd	24.2	nd	> 50	nd	45	nd	> 50	> 50
	k_2,_ s^−1^	0.2	19.0	nd	nd	6.3	7.1	9	18.4	5.9	8.9	11	16.6
37	k_1,_ s^−1^	> 50	nd	> 50	nd	> 50	nd	nd	nd	> 50	nd	> 50	nd
	k_2,_ s^−1^	0.2	20.5	1.9	20.4	12.0	9	nd	nd	7.3	12.3	18.9	31

k_1_ values corresponds to oxygen binding rate and k_2_ values to FeII‐O_2_ decay rate. Experiments with 4‐CF3‐phgua, 4‐Clphgua and 4‐MeOphgua analogues (not shown) were achieved and analysed with the same protocols as the ones described for 4‐Fphgua analogue (See Experimental procedures). nd, not determined.

Our results confirm the existence of three families of aryl‐guanidine that do not modify binding and coordination of dioxygen to iNOSoxy but that might affect Fe^II^‐O_2_ stability 37. Kinetic analysis of oxygen activation in the presence of BH_4_ supports our early hypothesis of the transient formation of an additional reaction intermediate 37. We then used RR spectroscopy coupled to fast‐kinetics methods to directly analyse the effect of aryl‐guanidine binding on dioxygen coordination and to look for potential new intermediates.

### Resonance Raman spectroscopy characterisation of NOS mechanism of hydroxylation

The nature of the intermediates formed in the course of Arg and Arg analogues oxidation have been analysed by resonance Raman spectroscopy using a home‐built T‐mixer set‐up 40 (see Experimental procedures). Solutions of reduced iNOSoxy in the presence of Arg or Arg analogues are mixed with ^16^O_2_‐ or ^18^O_2_‐saturated buffers and continuously pushed along a 250‐μm wide quartz tube. Focusing the RR laser beam at different distances from the mixing point allows determining the vibration modes of all species present in the reaction sample at a defined time (see Experimental procedures). We used two excitation wavelengths (413.1 and 441.6 nm) and recorded a spectral window that covers the 650–1600 cm^−1^ region. We focused on characteristic porphyrin modes and looked for the appearance of new bands that could reflect the O‐O stretch modes of Fe^II^‐O_2_ or other haem‐peroxo or hydroperoxo complexes. We used the kinetics data obtained by stopped‐flow to define the time of maximal Fe^II^‐O_2_ build‐up (see Experimental procedures).

**Figure 5:**
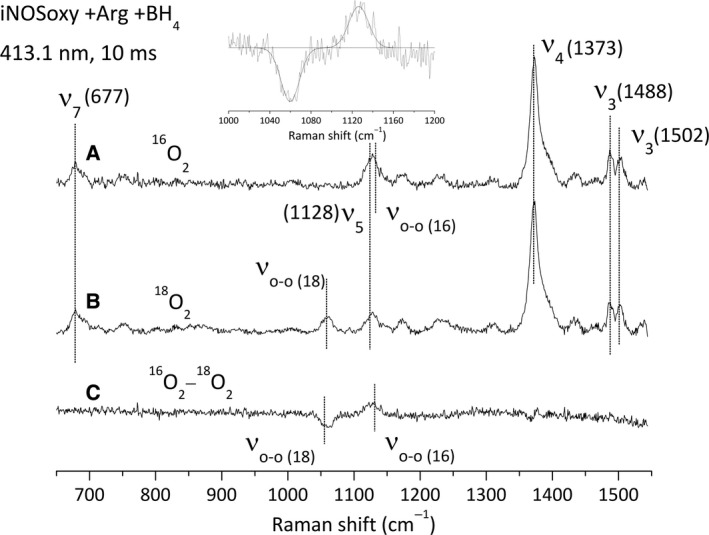
Resonance Raman spectra recorded at 413.1 nm of the Fe^II^O_2_ complex of iNOSoxy in the presence of Arg and BH_4_. (A) Resonance Raman spectrum of the Fe^II^O_2_ (^16^O_2_) complex. (B) Resonance Raman spectrum of the Fe^II^O_2_ (^18^O_2_) complex. (C) ^16^O_2_ minus ^18^O_2_ difference spectrum of Fe^II^O_2_ iNOSoxy complex (A minus B). The protein concentration used is about 40 μm after mixing and the spectra are recorded at 10 ms after mixing. Inset: simulation of the 1000–1200 cm^−1^ spectral region by a multigaussian function.

We first look to iNOSoxy‐catalysed oxidation of Arg (Fig. 5). The upper spectrum (Fig. 5A) shows the RR fingerprint of the sample for a 10 ms reaction time in the presence of ^16^O_2_. We observe a mixture of species with a minor fraction of the initial Fe^II^ species (bands ν_4_ at 1469 cm^−1^) and mostly ferric‐like complexes with characteristic bands at ν_7_ (677 cm^−1^), ν_5_ (1128 cm^−1^) and ν_4_ (1373 cm^−1^). Most of iNOSoxy seems to have reacted to form a mixture of Fe^III^ and Fe^II^‐O_2_ species with characteristic ν_3_ bands at 1488 cm^−1^ (Fe^III^) and 1502 cm^−1^ (Fe^II^‐O_2_) 29, 32. The Fe^II^‐O_2_ ν_O‐O_ is difficult to detect for it is expected around 1125 cm^−1^ 32 and is overlapping with the porphyrine mode ν_5_ (1122 cm^−1^). For this reason, we achieved the same experiment with a ^18^O_2_‐saturated buffer that results in an isotopic shift of ν_O‐O_ down to 1060 cm^−1^ but leaves ν_5_ unaffected (Fig. 5B). The difference between the ^16^O_2_ and the ^18^O_2_ spectra (Fig. 5C) specifically reveals the Fe^II^‐O_2_ ν_O‐O_ bands but not additional bands from other haem‐oxy species. Fitting of the ^16^O_2_/^18^O_2_ difference spectrum (see Experimental procedures) confirms the frequency of the ν_O‐O_ mode at 1127 (^16^O_2_) and 1060 cm^−1^ (^18^O_2_), which is similar to what has been reported previously 32 (Fig. 5 inset; Table 2). We achieved the same experiments using an additional excitation wavelength at 441.6 nm in ^16^O_2_ and ^18^O_2_ saturating conditions. We observed the same vibration modes for the Fe^II^‐O_2_ intermediate (data not shown) at similar frequencies (Table 2). Whatever the excitation wavelength or the O_2_‐saturating conditions, we did not observe any new intermediates such as Fe^III^‐OO^−^ or Fe^III^‐OOH during Arg oxidation reaction, for which the O‐O stretching mode would have been observed around 799 (^16^O_2_) and 755 (^18^O_2_) cm^−1^, values reported for cytochromes P450 complexes 44.

**Table 2:** Frequencies of the Raman lines of the dioxygen adducts observed with iNOSoxy and L‐Arg or Pentylgua or 4‐Fphgua

	λ_max_ (nm)	Time (ms)	ν_o‐o_ (^18^O_2_) (cm^−1^)	ν_o‐o_ (^16^O_2_) (cm^−1^)
Substrate‐free 33	413.1	2	1067	1133
Arg (pKa = 12.5)	413.1	10	1060	1127
Arg	441.6	10	1060	1127
Arg 33	413.1	2	1060	1126
Pentylgua (pKa = 12.6)	413.1	10	1062	1131
Pentylgua	441.6	40	–	–
Pentylgua	441.6	20	1061	1131
Pentylgua	441.6	4	1061	1130
4‐Fphgua (pKa = 10.8)	413.1	10	1064	1130
4‐Fphgua	441.6	10	–	–
4‐Fphgua	441.6	4	–	–
NOHA (pKa = 8.1) 33	413.1	2	1066	1132

–, not observed.

**Figure 6:**
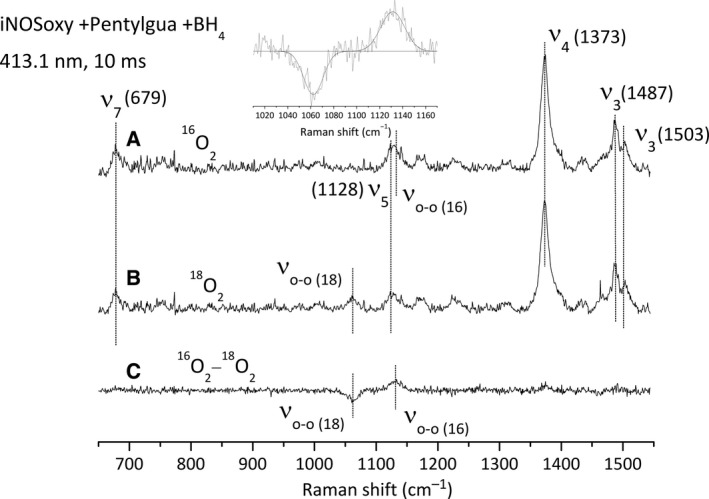
Resonance Raman spectra recorded at 413.1 nm of the Fe^II^O_2_ complex of iNOSoxy in the presence of Pentylgua and BH_4_. (A) Resonance Raman spectrum of the Fe^II^O_2_ (^16^O_2_) complex. (B) Resonance Raman spectrum of the Fe^II^O_2_ (^18^O_2_) complex. (C) ^16^O_2_ minus ^18^O_2_ difference spectrum of Fe^II^O_2_ iNOSoxy complex (A minus B). The protein concentration used is about 40 μm after mixing and the spectra are recorded at 10 ms after mixing. Inset: simulation of the 1010–1170 cm^−1^ spectral region by a multigaussian function.

We repeated the same series of experiments with ^16^O_2_ and ^18^O_2_ in the presence of BH_4_ and Pentylgua, an Arg analogue from Family 1 33. The RR fingerprints of the reaction sampled at 10 ms are displayed in Fig. 6. We observed porphyrin modes of the ferric species (Fig. 6, spectra A, B) at 677 cm^−1^ (ν_7_), 1122 cm^−1^ (ν_5_), 1373 cm^−1^ (ν_4_) and 1487 cm^−1^ (ν_3_). The ν_3_ mode of the Fe^II^‐O_2_ intermediate is observed at 1503 cm^−1^. The ^16^O_2_/^18^O_2_ difference spectrum shows the ν_O‐O_ mode in the ^16^O_2_ and ^18^O_2_ saturating conditions (Fig. 6, spectrum C). Fitting of the 1000–1200 spectral region (Fig. 6, inset) indicates ν_O‐O_ frequencies at 1131 (^16^O_2_) and 1062 cm^−1^ (^18^O_2_). These values are comparable but slightly higher than the ones reported in the presence of Arg. Likewise, the same experiments have been achieved using a 441.6‐nm excitation wavelength and at additional reaction times, that is, 4, 20 and 40 ms. We observe a maximum build‐up of the Fe^II^‐O_2_ intermediate at 4 ms, followed by a decrease in the intensity of the ν_O‐O_ bands leading to the complete disappearance of the Fe^II^‐O_2_ species at 40 ms (data not shown). The frequencies of the ν_O‐O_ modes are again similar to those determined at 413.1 nm (ν_O‐O(16)_ = 1130–1131 cm^−1^ and ν_O‐O(18)_ = 1061–1062 cm^−1^; Table 2)

**Figure 7:**
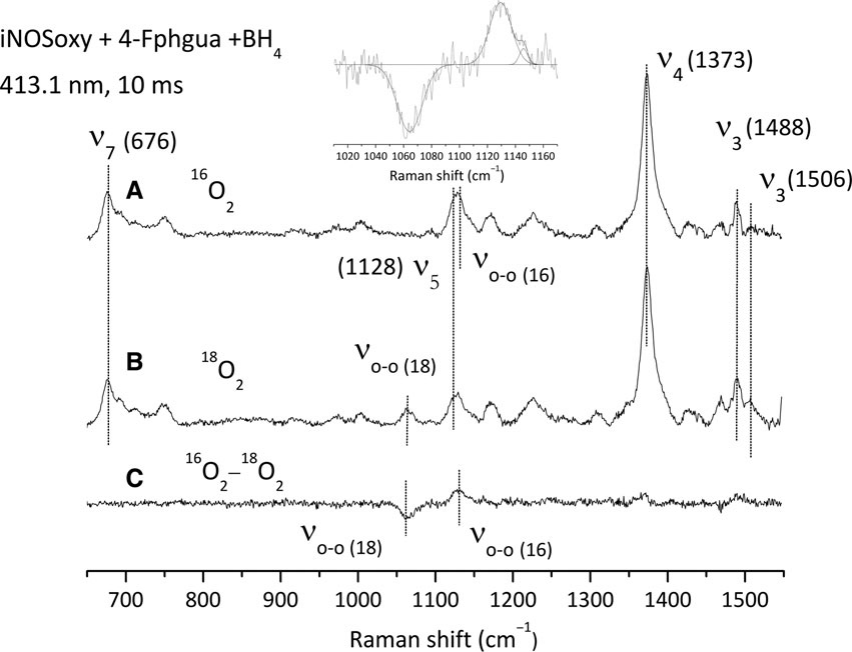
Resonance Raman spectra recorded at 413.1 nm of the Fe^II^O_2_ complex of iNOSoxy in the presence of 4‐Fphgua and BH_4_. (A) Resonance Raman spectrum of the Fe^II^O_2_ (^16^O_2_) complex. (B) Resonance Raman spectrum of the Fe^II^O_2_ (^18^O_2_) complex. (C) ^16^O_2_ minus ^18^O_2_ difference spectrum of Fe^II^O_2_ iNOSoxy complex (A minus B). The protein concentration used is about 40 μm after mixing and the spectra are recorded at 10 ms after mixing. Inset: simulation of the 1010–1170 cm^−1^ spectral region by a multigaussian function.

The last series of T‐mixer/RR experiment was achieved in the presence of BH_4_ and 4‐Fphgua, an Arg analogue from Family 2 for which no Fe^II^O_2_ intermediate has been observed by stopped‐flow 33. We observed the final ferric iNOSoxy modes at 677 cm^−1^ (ν_7_), 1122 cm^−1^ (ν_5_),1374 cm^−1^ (ν_4_) and 1488 cm^−1^ (ν_3_) with again a contribution at 1506 cm^−1^ that could be assigned to the ν_3_ mode of an Fe^II^O_2_ intermediate (Fig. 7, spectra A, B). The ^16^O_2_/^18^O_2_ difference spectrum (Fig. 7, spectrum C) reveals the presence of ν_O‐O_ vibrational mode in both the ^16^O_2_ and ^18^O_2_ conditions. The fitting of the 1000–1200 spectral region (Fig. 7, inset) leads to ν_O‐O_ frequencies at 1130 (^16^O_2_) and 1064 cm^−1^ (^18^O_2_). Unexpectedly, these modes were not observed using an excitation wavelength at 441.6 nm and did not allow either to identify other haem‐oxy intermediates (data not shown).

## Discussion

The characteristics of proton transfer process are a major topic to understand the mechanism of oxygen activation by NOS. Using a series of synthetic analogues of Arg, we recently proposed a model with the distal water molecule as potential second proton donor 33. The H‐bond established between this water molecule and the dioxygen ligand would be stabilized in the presence of Arg, but not NOHA. Thus, the nature of the distal H‐bond network appears as a key element in tuning ferric haem‐superoxo/peroxo reactivity and as such might control the balance between heterolytic cleavage (Step 1) and nucleophilic attack (Step 2) 13, 33, 36.

### A new approach to investigate oxygen activation mechanism

In this work, we wished to extend the model we deduced from the analysis of the structure of the Fe^II^‐CO complex 33 to the relevant Fe^II^‐O_2_ intermediate and establish a direct correlation between Fe^II^‐O_2_ structure/reactivity and its interaction with the distal H‐bond network. Following our first hypotheses 37, we aimed at identifying potential new haem‐oxy intermediates formed in the course of aryl‐guanidines oxidation. For this matter, we chose to combine the use of synthesised Arg analogues to an alternative and efficient fast‐kinetic approach, a T‐mixer device coupled to RR spectroscopy 40. Continuous flow analysis allows recording RR spectra for reaction times as small as a millisecond. These short‐time recordings might allow identifying short‐lived intermediates such as the Fe^II^‐O_2_ complex in the presence of Arg analogues. Besides, resonance Raman is an appropriate spectroscopy for characterising Fe^II^‐O_2_ structural properties and for identifying species that cannot be observed by UV–visible spectroscopy. We first furthered our initial stopped‐flow experiments in order to complete the set of kinetic parameters for single‐turnover oxidation reactions for various analogues of Arg (Table 1). Monitoring the absorbance at 428 nm during stopped‐flow analyses allows the estimation of the Fe^II^‐O_2_ build‐up and decay kinetics for the various oxidation reactions. It provides kinetic information that is essential to the trapping of reaction intermediates. We used two distinct excitation wavelengths (413.1 and 441.6 nm) to optimise the excitation and to reveal intermediates that exhibit different absorption profiles by RR spectroscopy. The frequencies determined for the porphyrin and ν_O‐O_ modes are independent from the excitation wavelength. The RR fingerprint of the reaction sampled regularly shows a mixture between the initial ferrous species, the final ferric species and the Fe^II^‐O_2_ intermediate which suggests that the chosen analysis time corresponds to the very middle of the reaction and is appropriate to trap Fe^II^‐O_2_ or any other haem‐oxy intermediates. We also analysed the reaction mixture at different times (4, 10, 20, 40 ms). We observed that all RR spectra showed the presence of the Fe^II^‐O_2_ species with an apparent maximum build‐up around 4 ms and a complete decay at 40 ms, which validates the choice of a reaction time at 10 ms. The method we set up proved to be appropriate since we were able to trap and characterise the Fe^II^‐O_2_ intermediate in the presence of aryl‐guanidines, which was not possible by UV–Visible absorption spectroscopy. The characterisation of the vibration modes of the Fe^II^‐O_2_ complex allowed us to investigate the interaction of Fe^II^‐O_2_ intermediate with its distal environment, and more particularly the H‐bond between the water molecule and the O_2_ distal ligand.

We determined the frequencies of the ν_O‐O_ modes of the Fe^II^‐O_2_ intermediate in the presence of Arg, pentylguanidine (Family 1) and 4‐Fphgua (Family 2). Frequencies are reported in Table 1 and show variations in the frequencies of the ν_O‐O_ mode as a function of the substrate bound in the pocket. The proximity between the frequencies of the ν_O‐O_ and the porphyrin ν_5_ modes could lead to a vibrational coupling between these vibrations. This coupling would correspond to a degeneration of the ν_O‐O_ and ν_5_ modes that would induce an enhancement of the porphyrin ν_5_ intensity and a shift of the ν_O‐O_ frequency towards ν_5_ frequency 45. For this reason, comparison among the analogues and substrates are discussed based on the ^18^O_2_ frequencies. In the presence of Pentylgua, the ν_O‐O_ frequency increases by 1–2 cm^−1^ (2–4 cm^−1^ in ^16^O_2_ conditions), which is reminiscent to what has been observed for the ν_C‐O_ mode of the Fe^II^‐CO complex 33, suggesting a weakening of the H‐bond between the water molecule and the dioxygen ligand. Although the arginine → Pentylgua substitution preserves the structure of the H‐bond (and the H‐bonding with the water molecule) changes in the guanidine structure might lead to a small destabilisation and weakening of this H‐bond, which is illustrated by the increased Fe^II^‐O_2_ autoxidation rate in the presence of Pentylgua. On the other hand, the Arg analogues from Family 2 are believed to modify the H‐bond distal network in a way similar to that observed for NOHA, that is, by displacing the water molecule away 33. This led to a strengthening of Fe^II^‐CO ν_C‐O_ stretch 33 (with respect to the absence of substrate), and likewise should induce an increase in ν_O‐O_ frequency toward values similar to the substrate‐free protein. Indeed, the ν_O‐O_ frequency increases by 6 cm^−1^ in the presence of NOHA (compared to Arg, Table 2). The same effect is observed in the presence of 4‐Fphgua, an aryl‐guanidine from Arg analogue Family 2, with an increase in 4 cm^−1^ of the ν_O‐O_ frequency. This supports our hypothesis concerning the effect of aryl‐guanidines on the distal H‐bond network: the pKa of these aryl‐guanidines (Family 2: 4‐Fphgua, 4‐CF_3_‐phgua, and 4‐Clphgua) is weaker than that of L‐Arg 33. This gives to the guanidinium proton a more acidic character, which in turn modifies the H‐bond network in such a way that the water molecule does not interact anymore with the ligand bound to the haem iron.

Our results show that despite its more linear coordination, CO remains a relatively good mimic of O_2_ to investigate O_2_ interactions with distal residues and reactivity. Globally, our results confirmed the correlation between the evolution of the vibration modes of the CO and O_2_ ligands that sense the changes in the distal environment and in the intensity of the electron back‐donation from iron orbitals. The role of NOS substrate in the proton transfer pathway and the nature of proton donor remains a matter of debate. Various reports from the groups of Rousseau 32, Crane 36, Marletta 38, Poulos 35 and from our groups 33, 37 support a model of NOS‐catalysed oxygen activation in which neither Arg nor NOHA directly act as the second proton donor. Instead, Arg would stabilise the distal H‐bond network in which the water plays a crucial role in the proton transfer sequence and in the formation of the oxoferryl intermediate (Step 1). In the presence of NOHA, the removal of the water molecule and the H‐bond between the distal oxygen and the substrate guanidinium stabilises the peroxo intermediate and prevents the Cpd I formation.

### Looking for new reaction intermediates

The second axis of our project was to look for reaction intermediates in NOS‐catalysed oxygen activation, using the T‐mixer/RR set‐up to reveal new species, in particular ferric haem‐peroxide that could not have been observed by UV–visible absorption spectroscopy. The initial hypothesis rose from the absence of Fe^II^‐O_2_ intermediate build‐up in the presence of BH_4_ and Family 2 aryl‐guanidines 37. This was in opposition with the observation of Fe^II^‐O_2_ build‐up, (a) in the presence of BH_2_ and (b) in the presence of BH_4_ and Family 3 analogues, both conditions for which no electron transfer is achieved 37. This suggests that Family 2 aryl‐guanidines do not prevent O_2_ binding. The fast Fe^II^‐O_2_ build‐up opposed to the slow Fe^III^ recovery, suggests the existence of an additional fast step converting Fe^II^‐O_2_ into an unknown intermediate that would slowly decay into the final Fe^III^ species (Fig. 4C). Since we were not able to identify this intermediate by stopped‐flow, we used the T‐Mixer/RR setup to trap and characterise it.

We were able to observe the Fe^II^‐O_2_ species in (almost) all conditions. We did not detect additional porphyrin or ν_O‐O_ bands, which suggested the absence of additional haem intermediate in the presence of Arg and Pentylgua (Family 1), but also with 4‐Fphgua, for which we previously hypothesised the transient formation of a haem‐peroxide complex. The nonobservation of bands specific to haem‐peroxide complex in the crowded 750–800 cm^−1^ spectral region might, however, be due to a weak build‐up of these intermediates (that would rapidly decompose into ferric species) and to the weak amplitude of their ν_O‐O_ bands.

The question of the absence of Fe^II^‐O_2_ build‐up in stopped‐flow experiment remains, leading to two alternative hypotheses: (a) the absorbance of iNOSoxy in the presence of BH_4_ and 4‐Fphgua might be blue‐shifted with an absorption maximum around 420 nm, such as what has been described for Oxy 1 species, a CYP‐like ferrous haem‐oxo species 30, 31. This is supported by the absence of RR exaltation of the Fe^II^‐O_2_ modes using an excitation wavelength at 441.6 nm, which also suggest a blue‐shift of Fe^II^‐O_2_ absorption. This shift would make the detection of Fe^II^‐O_2_ more difficult by optical absorption spectroscopy due to an absorption maximum too close to the Fe^II^ and Fe^III^ species. However, the BH_2_/4‐Fphgua Fe^II^‐O_2_ species displays a maximum absorption around 428 nm, and Family 3 aryl‐guanidines do not seem to modify this spectroscopic fingerprint. (b) The intermediate is not a haem‐oxy but a low‐spin ferric species with an absorption maximum around 415–420 nm. This intermediate could correspond to the produced hydroxy‐aryl‐guanidine that would be formed rapidly but would remain O‐bound to the haem iron 46, 47, which resulted in a Low‐Spin signature. It would be released slowly, with a rate corresponding to the ferric Low‐Spin/High‐Spin transition (Fig. 4B). However, our T‐Mixer/RR experiments did not show any low‐spin signals (apart from that assigned to Fe^II^‐O_2_) that could suggest the presence of such a Fe^III^ LS intermediate but instead reported the formation of a Fe^II^‐O_2_ species. At this stage, further experiments at longer reaction times are needed to favour any of these hypotheses.

### Conclusion

In this work we combined the organic synthesis of tailored substrate analogues with a fast‐kinetic approach that coupled a T‐Mixer module to resonance Raman spectroscopy: the T‐mixer allows investigating of short reaction times and RR is an alternative to optical spectroscopy for deciphering the nature of transient intermediates species. The experiments presented here allowed us to complete the structural characterisation of the Fe^II^‐O_2_ intermediate in the presence of various aryl‐ and alkyl‐substrate analogues. However, this advanced approach failed in identifying new intermediates in the oxygen activation reaction of NO‐Synthases. The peculiar absence of Fe^II^‐O_2_ build‐up in the presence of various aryl‐guanidines Arg analogues by optical spectroscopy remains unexplained so far and the proposed ferric haem‐peroxide species elude any characterisation. Nonetheless, our results confirm the role of the H‐bond network, in particular the acidity of the guanidinium and the interaction with the vicinal water molecule. This strengthens the scheme proposed for proton donation 13 that explains the difference between the first catalytic step (the water molecule allows the transfer of two protons and the formation of a [Fe^III^‐OOH_2_]^+^ intermediate) and the second catalytic step (no water molecule, one proton transfer leading to a Fe^III^‐OOH intermediate). This supports the model proposed for NOS molecular mechanism, for which Arg hydroxylation is related to the heterolytic cleavage of the [Fe^III^O‐OH_2_]^+^ bond and the build‐up of a Cpd I species unlike NOHA oxidation for which the Fe^III^‐OOH species is the oxidative intermediate.

## Author contributions

JS and MC planned experiments; AB, JL, JS and MC performed experiments; AB, JL, MC, JS analysed data; JLB contributed the analogues; AB, JL, PD, MC, JLB and JS wrote the paper.
